# Efficacy of traditional Chinese exercises in patients with post-COVID-19 chronic fatigue syndrome: A protocol for systematic review and meta-analysis

**DOI:** 10.1097/MD.0000000000031450

**Published:** 2022-11-18

**Authors:** Zhen Liu, Zhizhen Lv, Xingchen Zhou, Jiao Shi, Shuangwei Hong, Huazhi Huang, Lijiang Lv

**Affiliations:** a The Third Clinical Medical College of Zhejiang Chinese Medical University, Hangzhou, Zhejiang, China.

**Keywords:** anxiety, chronic fatigue syndrome, depression, post-COVID-19, traditional Chinese exercises

## Abstract

Some patients develop long-term symptoms after Corona virus disease 2019 (COVID-19), and chronic fatigue syndrome (CFS) is one of the main symptoms. CFS is characterized by fatigue lasting for more than 6 months accompanied by sleep disorders, anxiety, and depression, which causes a certain degree of harm to both physiological and psychological aspects of the individual. Traditional Chinese exercises (TCEs) are an ancient Chinese therapy and has recently been reported to be effective for CFS. Therefore, we will conduct a systematic review and meta-analysis aiming to accurately evaluate the efficacy of TCEs on post-COVID-19 CFS and provide an alternative treatment for post-COVID-19 CFS.

**Methods:**

Seven databases (PubMed, Ovid Embase, Cochrane Library, Web of Science, Chinese National Knowledge Infrastructure (CNKI), China Biology Medicine (CBM), and Wanfang) will be searched from establishment to August 2022, and we will only include randomized controlled trials of TCEs for post-COVID-19 CFS. Two reviews will independently include the research according to the inclusion and exclusion criteria. Review Manager 5.2 software will be used to analyze the accepted literature, and the relative risk ratio (RR) and 95% confidence interval (CI) will be used as effect indicators for the outcome indicator dichotomous variables. For continuous variables, weighted mean difference (MD) and 95% CI will be used as effect indicators. The heterogeneity test will be assessed using the *I*^2^ statistic and *Q* statistic. The PEDro scale was used to evaluate the methodological quality of the included studies. Subgroup analysis was performed according to different TCEs, age, gender, and duration of CFS.

**Results:**

This systematic review and meta-analysis will evaluate the efficacy of TCEs in post-COVID-19 CFS.

**Conclusion:**

The results of this study will provide reliable evidence for the effects of TCEs for post-COVID-19 CFS on patients’ fatigue, anxiety, depression, sleep, and quality of life.

## 1. Introduction

The novel Corona virus disease 2019 (COVID-19) pandemic has been lasting for nearly three years, a proportion of patients with mild to moderate COVID-19 develop long-term symptoms, and chronic fatigue syndrome (CFS) is one of the main symptoms.^[1–3]^ CFS, also known as myalgic encephalomyelitis, is a complicated chronic disease characterized by chronic fatigue lasting for more than 6 months. It is also accompanied by severe fatigue symptoms, depression, anxiety, and sleep disturbances, which seriously affects the quality of life of patients. what’s more, though the disease is not life-threatening, it may cause disability as well as rheumatoid arthritis and systemic lupus erythematosus.^[4–6]^ The prevalence of CFS is estimated to be between 1% and 2% globally, causing a serious burden on the work and life of patients.^[7,8]^ It is reported that at least 1 million people in the United States have CFS and that it is three to four times more common in women than in men, with the highest prevalence in people aged 40 to 50 years, but children and adolescents are also at risk.^[9]^ And it has a huge impact on the American economy, costing approximately $9 to 37 billion in lost productivity and $9 to 14 billion in direct medical costs annually.^[10,11]^ CFS is defined as a complex multisystem disease, and the pathogenesis of CFS is still unclear.^[12]^ In addition, due to the lack of biological disease markers, approximately 20 diagnostic criteria are available for CFS, and the symptoms of CFS can be easily confused with diseases such as the flu has led to a tendency to underdiagnose patients with CFS, which is a great test for consultation rates and patient compliance.^[13–15]^ Unfortunately, no specific medication is available to treat CFS, and only symptom relief is available. Considering the drug dependence and poor stability, more patients are turning to alternative therapies to relieve fatigue and improve quality of life.^[16]^

In China, Traditional Chinese exercises (TCEs) are widely used in the treatment of CFS. The exercises include Tai Chi, BaduanjJin, Yijinjing, Qigong, and Five-animal exercises, which play a major role in preventing and treating CFS.^[17]^ They can be used to unify the mind and body, eliminate fatigue and improve sleep quality through a wonderful combination of movements.^[18,19]^ They can also regulate the mood of CFS patients and provide better relief from anxiety and depression.^[20]^ Some studies have reported that TCEs have a positive effect on a patient’s physical function and mind, and the mechanism may be an increase in telomerase activity.^[21,22]^ Compared to common exercise therapy, TCEs may have similar health effects, but they are less intense and experience lower energy metabolism, which makes them more suitable for administration in patients with post-COVID-19 CFS.^[23]^ However, previous studies have only systematically reviewed partial TCEs for CFS, and insufficient evidence exists to support the effects of TCEs in improving fatigue, emotion, sleep, and quality of life in individuals with post-COVID-19 CFS.^[24,25]^ The evidence is still insufficient for TCEs in post-covid-19 CFS.

Therefore, the current study aimed to obtain a convincing conclusion as to whether TCEs contribute to a positive effect on fatigue, sleep, anxiety, depression, and quality of life in patients with CFS.

## 2. Methods

### 2.1. Study registration

This protocol of this systematic review strictly follows the PRISMA-P guidelines with a registration number (CRD42022361265) registered on the PROSPERO, and if necessary, we will keep our protocol constantly updated.^[26]^

### 2.2. Eligibility criteria

#### 2.2.1. Types of studies.

Only randomized controlled trials of TCEs for post-COVID-19 CFS will be included in this systematic review, and only randomized controlled trials in Chinese and English will be included.

#### 2.2.2. Types of participants.

First of all, the patients must have been previously infected with SARS-CoV-2 with typical symptoms and positive polymerase chain reaction test or antibodies without vaccination.^[27]^ And then, we will include the post-COVID-19 CFS patients who met either of the following diagnostic criteria: the Fukuda or Centers for Disease Control and Prevention criteria (CDC, 1994), Canadian Consensus Criteria (CCC, 2003), and the International Consensus Criteria (ICC, 2011).^[28–31]^

#### 2.2.3. Types of interventions and comparisons.

Post-COVID-19 CFS patients were treated by TCEs as the intervention group. The comparison groups involve other treatments, such as Common exercise, acupuncture, physiotherapy, and other therapies.

### 2.3. Types of outcome measures

#### 2.3.1. Primary outcomes.

The primary outcome was assessed by the recognized fatigue, anxiety, and depression scales: Fatigue Scale-14 (FS-14), Multidimensional Fatigue Inventory-20 (MFI-20), Hospital Anxiety and Depression Scale (HADS), Self-rating Depression Scale (SDS), and Self-Rating Anxiety Scale (SAS).

#### 2.3.2. Secondary outcomes.

The secondary outcomes will include quality of life, sleep, and anxiety. These items will also be assessed using following scales and neuropeptide substances: Neuropeptide Y (NPY), Calcitonin gene-related peptide (CGRP), the MOS item short form-36 health survey (SF-36), Pittsburgh Sleep Quality Index (PSQI).

### 2.4. Data sources

Seven databases (PubMed, Ovid Embase, Cochrane Library, Web of Science, Chinese National Knowledge Infrastructure (CNKI), China Biology Medicine (CBM), and Wanfang) will be searched from the beginning to August 2022 to choose RCTs that meet the requirements above. Table 1 shows the search strategy for the PubMed.

**Table 1:** Search strategy for PubMed database.

No.	Search items
#1	“Fatigue Syndrome, Chronic”[Mesh]
#2	“Chronic Fatigue Syndromes”[Title/Abstract] OR “Chronic Fatigue Syndromes”[Title/Abstract] OR “Chronic Fatigue Disorder”[Title/Abstract] OR “Fatigue Disorders, Chronic”[Title/Abstract] OR “fatigue”[Title/Abstract]
#3	#1 OR #2
#4	“post-acute COVID-19 syndrome” [Supplementary Concept]” OR “long-COVID”[Title/Abstract] OR “post-acute COVID syndrome”[Title/Abstract] OR “long COVID”[Title/Abstract] OR “chronic COVID syndrome”[Title/Abstract]
#5	#3 AND #4
#6	“traditional Chinese exercises”[Title/Abstract] OR “Tai Ji”[Mesh] OR “ Tai Chi”[Title/Abstract] OR “Tai Chi Chuan”[Title/Abstract] OR “Qigong”[Mesh] OR “Qi Gong”[Title/Abstract] OR “Baduanjin”[Title/Abstract] OR “Yijinjing”[Title/Abstract] OR “Five-animal exercises”[Title/Abstract] OR “Wuqinxi”[Title/Abstract]
#7	“randomized controlled trial”[Publication Type] OR “randomized”[Title/Abstract] OR “placebo”[Title/Abstract
#8	#3 AND #5 AND #7

### 2.5. Search strategy

#### 2.5.1. Study selection.

**Figure 1.**
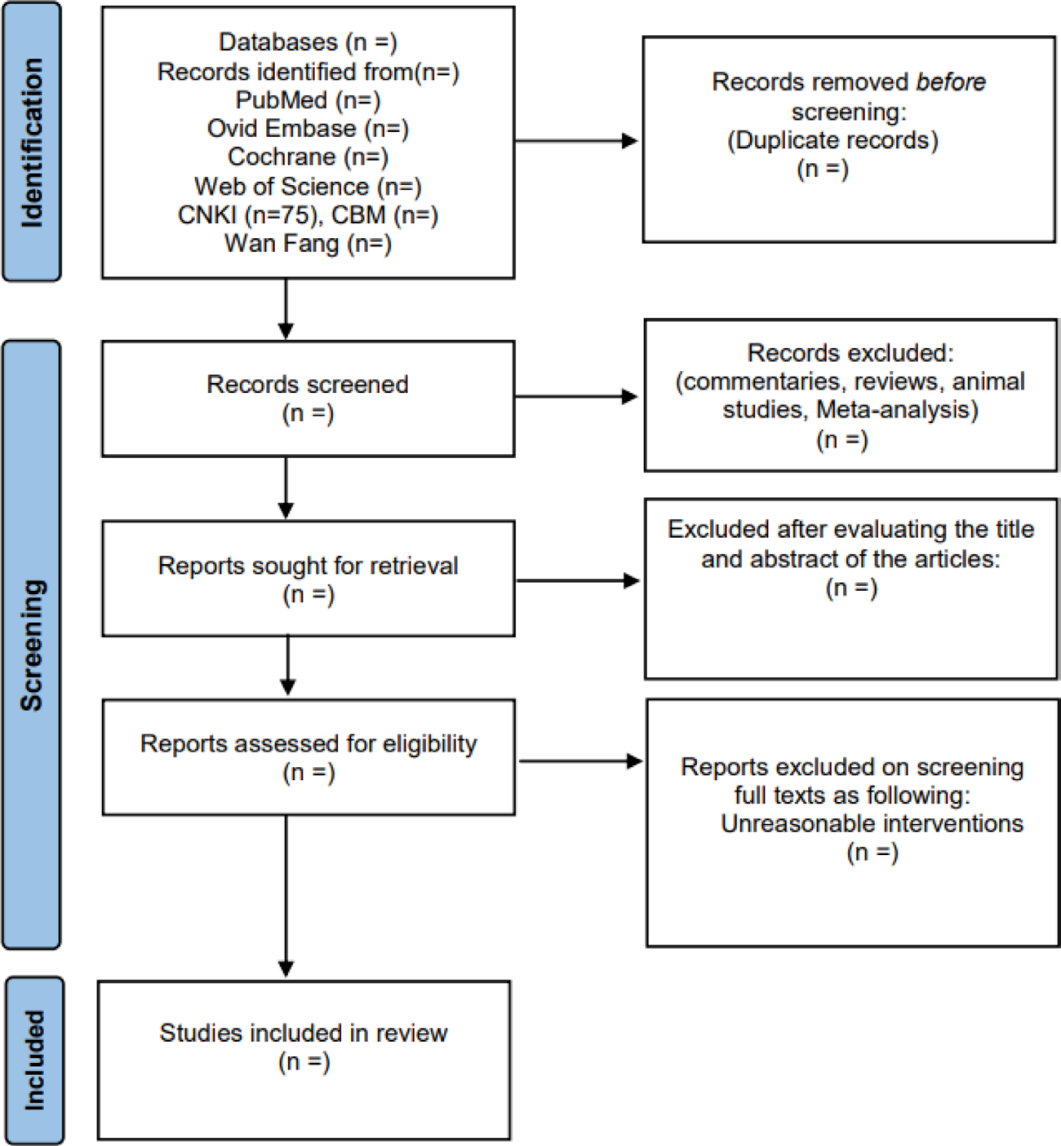
Flow chart for the review.

After eliminating duplicates from the retrieved research, two researchers will independently review the titles, abstracts, and eventually the full text according to the eligibility criteria. We will conduct a Cross-checking with each other after the screening was completed, and disagreements will be arbitrated by a third investigator. Figure 1 shows the process of the entire research selection.

#### 2.5.2. *Data extraction*.

Two reviewers (LZ, SJ) will jointly complete the data extraction. The following extracted data will be included: random assignment protocol, authors’ names, year of literature publication, number of included cases, age, interventions, control measures, treatment period, outcome indicators, diagnostic criteria, and dropouts. After checking, we fill in the data transformation into Review Manager 5.2, and disagreements will be arbitrated by a third investigator.

### 2.6. Quality evaluation

The quality of the RCTs will be assessed and scored by two reviewers using the Cochrane Risk of Bias Tool and the PEDro Scale. Biases in selection, performance, detection, attrition, reporting, and other aspects will be evaluated and classified as “low risk,” “high risk,” or “unclear,” the above are six items for the Cochrane bias risk tool. And the PEDro scale is composed of the following 11 items: predetermined research eligibility criteria, random patient assignment, allocation concealment, baseline data on between-group similarities, subject blinding, physical therapist blinding, assessor blinding, less than 15% dropouts, intention-to-treat analysis, comparison of between-group statistics, point measurement and variability data. Items 2 through 11 of the PEDro were assessed, resulting in a final score of 10 (item 1 was not scored).

### 2.7. Data synthesis

Review Manager 5.2 will be used to evaluate the selected research. We will use the relative risk ratio and 95% confidence interval (CI) to evaluate dichotomous data, and the standardized mean difference (MD) and 95% CI will be used to analyze continuous data. The heterogeneity test used *I*^2^ value and *Q* test as an effect indicator.^[32]^

#### 2.7.1. *Subgroup analysis.*

If high heterogeneity exists, we will perform subgroup analysis for sex, age, duration of exercise, duration of CFS, and different TCEs.

#### 2.7.2. Sensitivity analysis.

If the studies that will be included are of low quality or numerically far from other studies, we will conduct sensitivity analysis. Besides, we will iteratively remove one study at a time from Review Manager 5.2 to finish the analysis.

### 2.8. Ethics and dissemination

Systematic reviews don’t need ethics approval. Additionally, peer-reviewed publications will publish the current review’s findings.

## 3. Discussion

It is reported that COVID-19 pandemic contributes to an increase in CFS patients.^[33]^ Since viral infections are frequently the cause of CFS, also since about 30% of individuals who don’t fully recover from the acute phase of COVID-19 suffer from the condition known as the post-COVID-19 syndrome, which is characterized by fatigue intolerance, persistent fatigue, insomnia and anxiety.^[34–36]^ TCEs are painless therapies inherited from China and have high safety. It has been proven in a randomized controlled trial that TCEs can relieve fatigue and insomnia in CFS, but no systematic review of TCEs for post-COVID-19 CFS is currently available.^[20]^ Therefore, the present systematic review and meta-analysis will evaluate the evidence of fatigue, anxiety, depression, sleep, and quality of life in TCEs for post-COVID-19 CFS. The results of the study will provide patients with the option of pain-free treatment for post-COVID-19 CFS. Importantly, the financial burden on patients will be greatly alleviated.

## Author contributions

**Conceptualization:** Zhen Liu.

**Data curation:** Jiao Shi, Huazhi Huang, Shuangwei Hong, Zhen Liu, Zhizhen Lv.

**Formal analysis:** Zhen Liu, Zhizhen Lv.

**Funding acquisition:** Lijiang Lv.

**Investigation:** Lijiang Lv, Zhizhen Lv.

**Methodology:** Jiao Shi, Xingchen Zhou, Zhen Liu.

**Supervision:** Lijiang Lv.

**Writing – original draft:** Zhen Liu.

**Writing – review & editing:** Zhen Liu, Zhizhen Lv.
